# Incidence of sepsis and associated mortality within the first year after cancer diagnosis in middle aged adults: A US population based study

**DOI:** 10.1371/journal.pone.0243449

**Published:** 2020-12-28

**Authors:** Andry Van de Louw, Austin Cohrs, Douglas Leslie

**Affiliations:** 1 Division of Pulmonary and Critical Care Medicine, Penn State Health Milton S Hershey Medical Center, Hershey, Pennsylvania, United States of America; 2 Department of Public Health Sciences, Penn State Health Milton S Hershey Medical Center, Hershey, Pennsylvania, United States of America; Flinders Medical Centre, AUSTRALIA

## Abstract

**Background:**

The incidence of sepsis has been rising overall but updated data in cancer patients are lacking. After a cancer diagnosis, incidence of sepsis and overall mortality peak within the first year. However, how much sepsis contributes to mortality remains unclear. We used a multistate model approach to analyze the incidence, risk factors and associated mortality of sepsis within 1 year of cancer diagnosis in middle aged adults.

**Methods:**

Analysis of a large US health insurance claims database (Marketscan) between 2005 and 2014. Patients with a new diagnosis of cancer who received chemotherapy were included. Within a year of diagnosis, we assessed inpatient admissions for sepsis based on ICD-9 codes and survival using hospitalizations, outpatient visits and prescriptions filled. Competing risk and multistate models were used to assess the incidence of sepsis and transition probabilities between cancer, sepsis and death.

**Results:**

119,379 patients (38.9% males), aged 55 (50–60) years, were included; 2,560 developed isolated sepsis, 477 severe sepsis and 1331 septic shock within 1 year, with associated hospital mortality of 14.8%, 30% and 46% respectively. The probability of sepsis increased between 2005 and 2014; at 1 year, its cumulative incidence was 3.7% with a probability of mortality after sepsis of 35.5% (95% CI 21.6%-50.9%). Age, male gender, Charlson comorbidity index, hematological malignancies and metastases at diagnosis were associated with sepsis and mortality.

**Conclusions:**

Incidence and mortality of sepsis were 3.7% and 35.5% at 1 year after cancer diagnosis and were both associated with baseline patient and cancer characteristics.

## Introduction

Cancer is a major public health concern with estimates of 18.1 million new cases and 9.6 million cancer deaths worldwide in 2018 [1]. It is the second leading cause of death in the United States after cardiovascular diseases [2]. Sepsis is another public health issue and although various definitions and reporting make its incidence difficult to ascertain, worldwide estimates as high as 31.5 million cases and 5.3 million deaths have been reported [3]. Both cancer and sepsis have been the focus of intense preventive efforts [4, 5], however large epidemiological studies on sepsis in cancer patients are scarce.

Two studies performed 2–3 decades ago reported incidences of sepsis of about 1,500–1,600 cases per 100,000 cancer patients per year in the United States [6, 7]. Cancer patients have a relative risk of about 4 for severe sepsis as compared to the general population [6].

More recent epidemiological data are necessary for two reasons: first, recent studies suggest that the mortality of critically ill cancer patients with sepsis has decreased over time [8]. Second, the incidence of sepsis has been steadily increasing in the general population [9] and also probably in cancer patients: a recent study reported that the incidence of sepsis in cancer patients reached 6.4% within a year [10]. The net result in cancer patients of the increased incidence and decreased mortality of sepsis observed over the past 2 decades is unknown. The incidence of sepsis seems to peak during the first year after cancer diagnosis [10] probably due to the higher treatment intensity (surgery, chemotherapy); standardized mortality ratios [11] also peak within 1 year, but how much sepsis contributes to this mortality is unclear, as no study has investigated sepsis as a time dependent variable with non-sepsis related death as a competing event. The objective of this study was to update the data on sepsis incidence, to investigate its risk factors (demographics, comorbidities, baseline cancer characteristics) and its contribution to overall mortality within the first year of cancer diagnosis in middle age adults, using competing risk and multistate models.

## Methods

This study was approved by the Penn State Health Milton S Hershey Medical Center institutional review board (study number 6364). Informed consent was waived as data analyzed were extracted from a data registry and were deidentified. We used 2005–2014 data from the Truven Health MarketScan database. The database is a commercially available health insurance claims database. It includes claims data for a sample of more than 245 million privately insured people in all 50 US states, including demographic characteristics, health care utilization and costs, dates of service, diagnosis codes and procedure codes. The data represent claims from clinicians, hospitals, and pharmacies that have been adjudicated for payment and are obtained directly from a sample of large employers and health plans. Marketscan does not include patients covered by Medicare, a U.S. federal government program primarily providing health insurance for Americans aged 65 and older. Truven Health has a quality-control process to verify that the data meet criteria for quality and completeness and Marketscan has been used in multiple other studies, including studies examining complications and follow-up care after health care procedures [12, 13].

All patients in the database who met the following criteria were included: 1) age greater than or equal to 40 years; 2) diagnosis of cancer between 2006 and 2014 based on ICD-9 codes: 140–149.9 for oral and pharyngeal cancers, 176–176.9 for gastrointestinal system, 176–176.9 for respiratory system, 176–176.9 for musculoskeletal and breast cancers, 179–189.9 for genitourinary system, 190–199.9 for other and unspecified sites, 200–209.9 for hematological malignancies, 235–238.9 for cancers of uncertain behavior, 239–239.9 for cancers of unspecified nature; 3) to include only patients with new cancer diagnoses, patients had to be continuously enrolled in the database, without a diagnosis code for cancer or a procedure code for chemotherapy or radiotherapy, for at least 12 months prior to the index date of cancer diagnosis; 4) administration of chemotherapy within 6 months of the index date of cancer diagnosis, based on either one of ICD-9 CM codes of 99.25, V58.1x, V66.2, V67.2, CPT-4 codes of 96400–96549, J9000-J9999, Q0083-Q0085, revenue center codes of 0331, 0332, and 0335; 5) to ensure a minimal follow-up, patients had to be continuously enrolled in the database at least 12 months after the index date of cancer diagnosis, unless they died.

Comorbidities were assessed by screening the database during 1 year prior to cancer diagnosis for any ICD-9 diagnosis included in the Charlson comorbidity index (CCI) and the CCI was then computed with all its components.

All included patients were screened for hospital admissions with ICD-9 diagnoses of sepsis (995.91), severe sepsis (995.92) or septic shock (785.52) within the first year after cancer diagnosis. Sepsis, severe sepsis and septic shock represent 3 syndromes associated with gradually increasing mortality: sepsis is defined by a proven or suspected infection with signs of systemic inflammation in response to infection [14] or systemic inflammatory response syndrome [15], severe sepsis is defined by sepsis with organ dysfunction (for instance encephalopathy, acute kidney injury) and septic shock is defined by sepsis associated with persistent hypotension despite adequate volume resuscitation [14]. By definition, patients with severe sepsis or septic shock have sepsis and throughout the manuscript sepsis will be defined by the presence of at least one of these 3 ICD-9 codes unless otherwise specified.

For these admissions, ICD-9 principal and secondary diagnoses (up to 15 per admission), procedures codes (up to 15 per admission) and discharge status were collected. For patients with multiple admissions for sepsis, only the first one was taken into account to ensure independence of observations.

In order to assess 1-year survival, we screened follow-up information available in the database; survival status at 1 year was defined based on discharge status for inpatient admissions (regardless of diagnoses), physician office visits and outpatient prescription fillings.

### Statistical analysis

The r statistical package version 3.6.1 was used for statistical analysis. Data are presented as median (interquartile range) and number (percentage) for continuous and categorical variables respectively. Baseline characteristics of patients with and without sepsis within 1 year of cancer diagnosis were compared with Wilcoxon rank sum test or chi-square test as appropriate. The cumulative incidence of sepsis was analyzed using a competing risk approach and subdistribution hazards (SDH) were computed with a Fine-Gray model, with death without sepsis treated as a competing risk (package comprsk). Age, gender, CCI, cancer site and metastases were the covariates included in all models. Sepsis was included as a time-dependent variable in a Cox model to assess its impact on 1-year mortality. The proportional hazards assumptions were checked by visual inspection of the Schoenfeld residuals plots. In order to assess the effect of covariates on the probability of sepsis and 1-year mortality we also used a multistate model including 3 states: cancer (baseline state for all patients), sepsis and death (packages p3state and TP.msm). As the Markov assumption was not fulfilled we modeled the effect of covariates with a Cox semi-Markov model and we used Kaplan-Meier weighted estimates with 95% bootstrap confidence intervals to compute transition probabilities between the three states. p<0.05 was considered for statistical significance.

## Results

**Fig 1:**
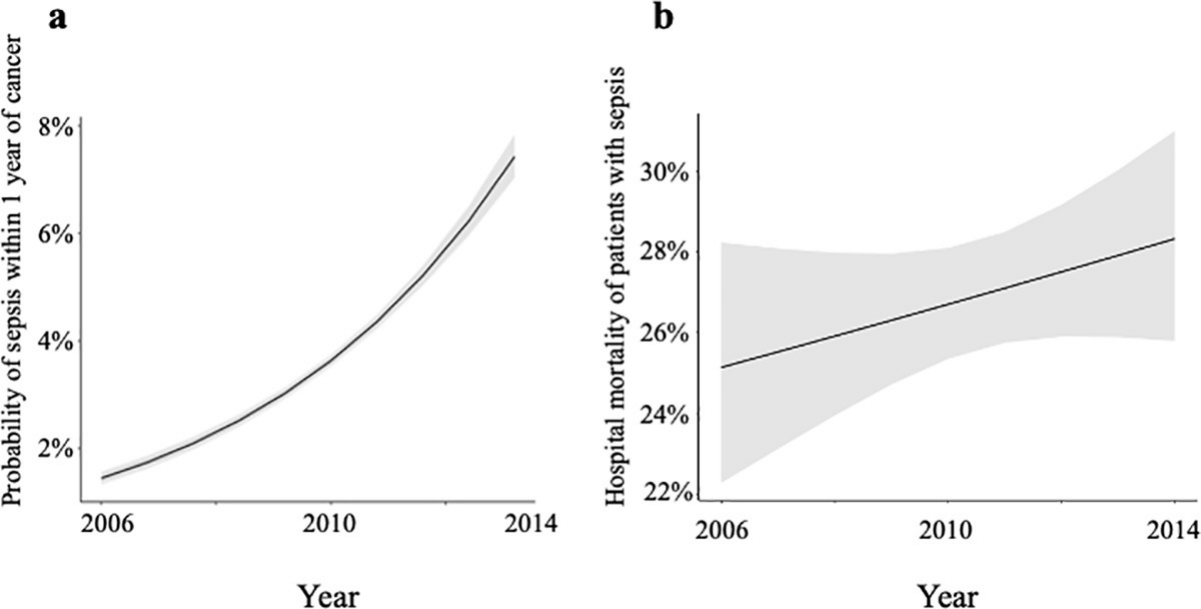
Time course over the study period (2006–2014) of unadjusted probability of sepsis within 1 year for patients with a new cancer diagnosis (Fig 1(A)) and unadjusted hospital mortality for the subset of patients admitted with sepsis (Fig 1(B)). Black lines represent the unadjusted probabilities and grey areas represent the 95% confidence intervals.

The study included 119,379 patients (38.9% males), aged 55 (50–60) years with baseline CCI of 0 (67.6%), 1 or 2 (28.8%), 3 or 4 (2.8%) or above 5 (0.8%). Cancer site was distributed as follows: hematological malignancy (9%), digestive (16%), genitourinary (14%), head and neck (2%), muskuloskeletal and breast (31%), respiratory (8%) and other (20%). About 11% of patients had metastatic disease at diagnosis. The main characteristics of the patients, based on cancer site, are detailed in Table 1, and univariate comparison between patients who did and did not develop sepsis is reported in Table 2. Overall, 4368 patients (3.7%) were hospitalized with a diagnosis of sepsis within 1 year of cancer diagnosis: 890 (20%) of these patients had neutropenia, 2,560 (59%) had sepsis without severe sepsis or septic shock, 477 (11%) severe sepsis and 1331 (30%) septic shock. The hospital mortality was 14.8%, 30% and 46% for patients with sepsis without severe sepsis or septic shock, severe sepsis and septic shock respectively (p<0.001). The CCI of patients with sepsis who died was 0 (52%), 1 or 2 (38%), 3 or 4 (6%) and above 5 (4%) and was not different from the CCI of patients with sepsis who survived (p = 0.38). Unadjusted probability of sepsis and hospital mortality over the study time period are displayed in Fig 1. the incidence of sepsis increased between 2006 and 2014 whereas hospital mortality tended to increase as well, but less strikingly.

**Table 1:** Characteristics of the 119,379 patients with cancer included in the study between 2005 and 2014, based on specified cancer sites.

Cancer site	Patients, n (%)	Males, n (%)	Age	CCI 1–2,	CCI >2,	Metastases at diagnosis,	Sepsis within 1 year,	Time of sepsis (days)
			(years, IQR)	n (%)	n (%)	n (%)	n (%)	
**Bladder**	2212 (1.9%)	1728 (78.1%)	58 (54–61)	702 (32%)	88 (4%)	14 (0.6%)	64 (3%)	189 (79–246)
**Bone**	239 (0.2%)	138 (57.7%)	55 (49–59)	59 (25%)	9 (4%)	13 (5%)	10 (4%)	152 (84–230)
**Brain**	596 (0.5%)	336 (56.4%)	54 (48–59)	164 (28%)	23 (4%)	12 (2%)	27 (5%)	102 (78–139)
**Breast**	33420 (28%)	235 (0.7%)	53 (47–58)	6694 (20%)	424 (1%)	1349 (4%)	400 (1%)	118 (78–179)
**Colorectal**	10589 (8.9%)	5650 (53.4%)	55 (50–59)	2751 (26%)	296 (3%)	1345 (13%)	387 (4%)	130 (61–230)
**Connective / soft tissue**	402 (0.3%)	214 (53.2%)	54 (48–59)	110 (27%)	16 (4%)	24 (6%)	21 (5%)	158 (98–247)
**Ear / nose**	103 (0.1%)	69 (67.0%)	54 (48–58)	18 (18%)	3 (3%)	1 (1%)	5 (5%)	161 (103–182)
**Esophagus**	1379 (1.2%)	1162 (84.3%)	57 (53–61)	477 (35%)	55 (4%)	44 (3%)	111 (8%)	139 (82–222)
**Hepatobiliary**	1505 (1.3%)	919 (61.1%)	57 (53–60)	750 (50%)	323 (2%)	114 (8%)	165 (11%)	129 (59–201)
**Hodgkin lymphoma**	759 (0.6%)	453 (59.7%)	50 (45–56)	199 (26%)	45 (6%)	N/A	35 (5%)	123 (52–198)
**Kidney**	576 (0.5%)	395 (68.6%)	57 (52–60)	214 (37%)	30 (5%)	83 (14%)	28 (5%)	112 (45–189)
**Larynx**	450 (0.4%)	342 (76.0%)	57 (53–60)	154 (34%)	28 (6%)	17 (4%)	17 (4%)	109 (93–250)
**Lung**	7839 (6.6%)	4032 (51.4%)	58 (53–61)	3726 (48%)	487 (6%)	1016 (13%)	404 (5%)	121 (57–218)
**Lymph nodes metastases,unspecified**	2207 (1.8%)	898 (40.7%)	55 (50–59)	629 (29%)	61 (3%)	N/A	63 (3%)	113 (76–184)
**Lymphoid leukemia**	715 (0.6%)	454 (63.5%)	55 (49–59)	206 (29%)	23 (3%)	N/A	74 (10%)	131 (55–217)
**Melanoma**	499 (0.4%)	290 (58.1%)	54 (48–59)	109 (22%)	12 (2%)	13 (3%)	6 (1%)	35 (27–86)
**Mouth / pharynx**	1730 (1.4%)	1394 (80.6%)	56 (51–60)	453 (26%)	61 (4%)	139 (8%)	71 (4%)	105 (66–204)
**Multiple myeloma**	1746 (1.5%)	984 (56.4%)	56 (51–60)	622 (36%)	158 (9%)	N/A	127 (7%)	144 (77–220)
**Myeloid leukemia**	1033 (0.9%)	546 (52.9%)	54 (48–60)	325 (32%)	34 (3%)	N/A	189 (18%)	89 (23–172)
**Neuroendocrine**	215 (0.2%)	108 (50.2%)	55 (50–59)	88 (41%)	9 (4%)	24 (11%)	11 (5%)	85 (52–166)
**Non Hodgkin lymphoma**	5224 (4.4%)	3001 (57.4%)	55 (49–59)	1590 (30%)	278 (5%)	N/A	269 (5%)	95 (44–179)
**Ovary**	2896 (2.4%)	10 (0.3%)	55 (49–59)	700 (24%)	60 (2%)	723 (25%)	90 (3%)	94 (37–176)
**Pancreas**	1829 (1.5%)	1009 (55.2%)	57 (53–61)	859 (47%)	116 (6%)	331 (18%)	201 (11%)	118 (50–213)
**Prostate**	6186 (5.2%)	6179 (99.9%)	59 (56–62)	1796 (29%)	230 (4%)	60 (1%)	56 (1%)	94 (12–182)
**Respiratory / GI metastases**	2112 (1.8%)	930 (44.0%)	57 (51–60)	902 (43%)	101 (5%)	N/A	158 (7%)	105 (49–219)
**Retroperitoneum / peritoneum**	285 (0.2%)	61 (21.4%)	57 (51–60)	87 (31%)	6 (2%)	52 (18%)	17 (6%)	93 (32–229)
**Small intestine**	281 (0.2%)	156 (55.5%)	55 (49–59)	107 (38%)	13 (5%)	54 (19%)	24 (9%)	133 (36–225)
**Stomach**	1127 (0.9%)	774 (68.7%)	56 (50–60)	424 (38%)	58 (5%)	122 (11%)	90 (8%)	168 (99–252)
**Testis**	281 (0.2%)	280 (99.6%)	47 (43–52)	59 (21%)	9 (3%)	14 (5%)	7 (2%)	143 (98–174)
**Thymus/heart/mediastinum**	169 (0.1%)	90 (53.3%)	55 (49–59)	70 (41%)	8 (5%)	14 (8%)	12 (7%)	87 (63–193)
**Thyroid**	202 (0.2%)	68 (33.7%)	54 (48–58)	82 (41%)	6 (3%)	7 (3%)	4 (2%)	111 (89–160)
**Uterus**	3492 (2.9%)	7 (0.2%)	56 (50–60)	933 (27%)	54 (2%)	167 (5%)	113 (3%)	136 (72–262)

CCI: Charlson comorbidity index.

**Table 2:** Univariate analysis comparing cancer patients who did and did not develop sepsis within 1 year of cancer diagnosis.

	No sepsis	Sepsis	p value
	(n = 115011)	(n = 4368)	
**Age (years)**	55 (49–59)	57 (51–60)	< 0.001
**Male gender, n (%)**	44199 (38.4%)	2248 (51.5%)	< 0.001
**CCI 1–2, n (%)**	32731 (29.1%)	1647 (38.8%)	< 0.001
**CCI >2, n (%)**	3884 (3.5%)	348 (8.2%)	< 0.001
**Metastases at diagnosis, n (%)**	12064 (10.5%)	718 (16.4%)	< 0.001
**Myocardial infarction, n (%)**	891 (0.8%)	46 (1.1%)	0.037
**Congestive heart failure, n (%)**	1539 (1.4%)	126 (3.0%)	< 0.001
**Peripheral vascular disease, n (%)**	2815 (2.5%)	185 (4.4%)	< 0.001
**Cerebrovascular disease, n (%)**	3123 (2.8%)	180 (4.2%)	< 0.001
**Dementia, n (%)**	41 (<0.1%)	1 (<0.1%)	0.663
**Chronic pulmonary disease, n (%)**	12049 (10.7%)	635 (15.0%)	< 0.001
**Rheumatic disease, n (%)**	2327 (2.1%)	104 (2.4%)	0.090
**Mild liver disease, n (%)**	5634 (5.0%)	427 (10.1%)	< 0.001
**Diabetes without chronic complication, n (%)**	16217 (14.4%)	908 (21.4%)	< 0.001
**Diabetes with chronic complication, n (%)**	2297 (2.0%)	168 (4.0%)	< 0.001
**Hemiplegia or paraplegia, n (%)**	216 (0.2%)	15 (0.4%)	0.021
**Renal disease, n (%)**	1672 (1.5%)	167 (3.9%)	< 0.001
**Moderate or severe liver disease, n (%)**	372 (0.3%)	43 (1.0%)	< 0.001
**AIDS / HIV, n (%)**	362 (0.3%)	40 (0.9%)	< 0.001
**Cancer site, n (%)**			< 0.001
***digestive***	17751 (15.4%)	1108 (25.4%)	
***genitourinary***	16211 (14.1%)	395 (9.0%)	
***head and neck***	2091 (1.8%)	87 (2.0%)	
***hematological malignancy***	10049 (8.7%)	796 (18.2%)	
***Musculoskeletal/breast***	36786 (32.0%)	512 (11.7%)	
***other site***	23219 (20.2%)	987 (22.6%)	
***respiratory system***	8904 (7.7%)	483 (11.1%)	

CCI: Charlson comorbidity index.

**Fig 2:**
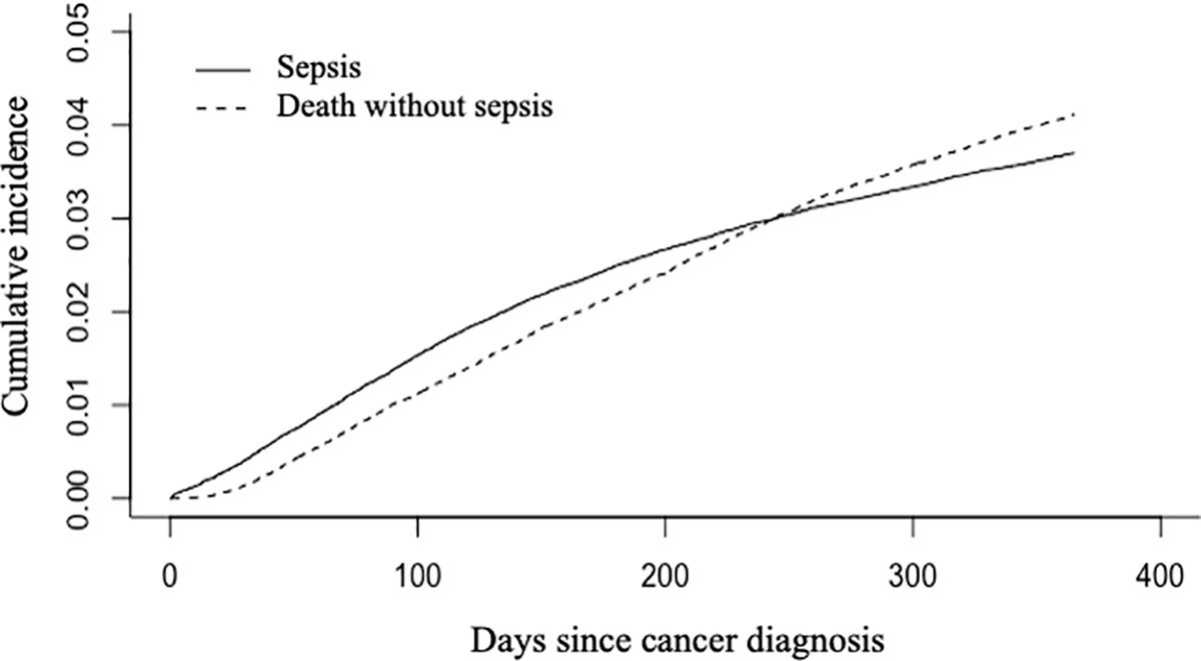
Cumulative incidence of sepsis within the first year after cancer diagnosis in a cohort of 119,379 patients, with death without sepsis treated as a competing event.

The cumulative incidence of sepsis was 0.4%, 1.4%, 2.5% and 3.7% at 1, 3, 6 and 12 months respectively (Fig 2). Age, CCI and hematological malignancies were associated with increased hazard for sepsis whereas female gender and other cancer sites (as compared to digestive) were associated with decreased hazard (Table 3).

**Table 3:** Effect of baseline characteristics on the subdistribution hazard (SDH) of sepsis within 1 year after cancer diagnosis, based on a competing risk model with death without sepsis treated as the competing risk.

Variable	Fine and Gray model	
	SDH (95% CI)	p
**Age (years)**	1.02 (1.02–1.03)	<0.001
**Female gender**	0.92 (0.87–0.98)	0.01
**Charlson comorbidity index**	1.22 (1.19–1.24)	<0.001
**Cancer site (reference: digestive)**		
*Genitourinary*	0.41 (0.36–0.46)	<0.001
*Head and neck*	0.67 (0.54–0.83)	<0.001
*Hematological malignancy*	1.34 (1.22–1.47)	<0.001
*Musculoskeletal/breast*	0.27 (0.24–0.31)	<0.001
*Respiratory*	0.81 (0.73–0.90)	<0.001
**Metastases at diagnosis**	1.65 (1.51–1.79)	<0.001

**Fig 3:**
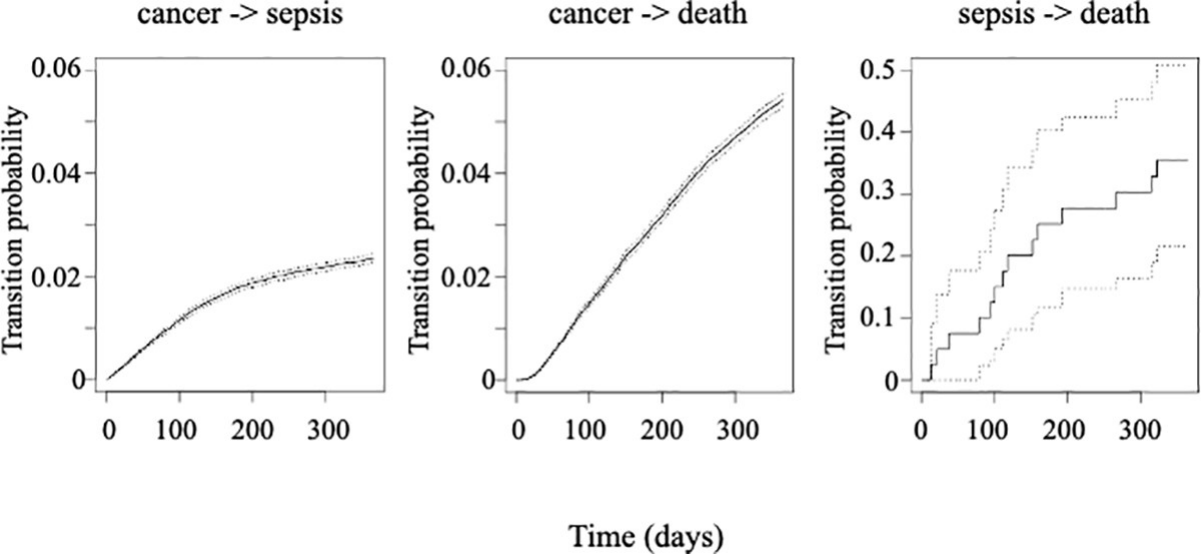
Transition probabilities within the first year after cancer diagnosis between the states « cancer », « sepsis » and « death » based on the multistate model. Dark lines represent for a patient diagnosed with cancer at day 0 the probability of transitioning from one state to another over time; grey dotted lines represent the 95% confidence intervals. Cancer->death represents the transition from cancer to death without sepsis.

After adjustment for age, gender, CCI, cancer site and metastases, sepsis was strongly associated with 1-year mortality in the time-dependent Cox model (HR 10.28, 95% CI 9.73–10.85, p<0.001). In a sensitivity analysis excluding patients with severe sepsis or septic shock, sepsis alone remained associated with 1-year mortality (HR 6.27, 95% CI 5.80–6.78, p<0.001). Within a year of cancer diagnosis, the transition probabilities were 2.4% (95% CI 2.3%-2.4%) for cancer to sepsis, 5.4% (95% CI 5.3%-5.6%) for cancer to death without sepsis and 35.5% (95% CI 21.6%-50.9%) for sepsis to death. Fig 3 displays the hazards for transitioning between the 3 states within the first year: the hazard for sepsis was higher than the hazard for death without sepsis within the first 60 days when the two curves crossed, with hazard for death without sepsis then steadily rising while the slope of the rise decreased for hazard of sepsis. Female gender was associated with decreased transition intensities for all state transitions (cancer to sepsis, cancer to death without sepsis and sepsis to death) whereas age, CCI, cancer site and metastatic disease were all associated with increased transition intensities between all states (Table 4). CCI and metastases at diagnosis were the variables most strongly associated with transition to sepsis and death without sepsis whereas metastatic disease but not CCI was the strongest predictor of transition from sepsis to death (Table 4).

**Table 4:** Summary of the multistate Cox semi-markov model assessing the effect of baseline characteristics on the transition between cancer, sepsis and death.

Variable	Hazard ratio (95% CI)	p
	**Transition intensity from cancer to death (without sepsis)**	
**Age (years)**	1.03 (1.02–1.03)	<0.001
**Female gender**	0.56 (0.54–0.59)	<0.001
**Charlson comorbidity index**	1.23 (1.22–1.26)	<0.001
**Cancer site**	1.10 (1.08–1.11)	<0.001
**Metastases at diagnosis**	2.73 (2.59–2.89)	<0.001
	**Transition intensity from cancer to sepsis**	
**Age (years)**	1.02 (1.02–1.03)	<0.001
**Female gender**	0.67 (0.64–0.70)	<0.001
**Charlson comorbidity index**	1.30 (1.28–1.32)	<0.001
**Cancer site**	0.96 (0.95–0.97)	<0.001
**Metastases at diagnosis**	1.87 (1.75–1.99)	<0.001
	**Transition intensity from sepsis to death**	
**Age (years)**	1.03 (1.02–1.03)	<0.001
**Female gender**	0.86 (0.79–0.93)	<0.001
**Charlson comorbidity index**	1.03 (1.00–1.06)	0.055
**Cancer site**	1.03 (1.02–1.05)	<0.001
**Metastases at diagnosis**	1.51 (1.37–1.68)	<0.001

Table 5 summarizes the infectious sites and agents among the 4,368 patients who developed sepsis: the most frequent infections were bloodstream infections and pneumonias, each involving about a third of patients, and gram-negative rods (mostly Escherichia coli and Pseudomonas spp) and gram-positive cocci (staphylococcus spp followed by streptococcus spp) each accounted for about half of the documented bacteria.

**Table 5:** Infection sites and infectious agents for the 4368 patients who developed sepsis within 1 year of cancer diagnosis.

**Infection site**	**n (%)**
Unspecified septicemia	2817 (64)
Bacteremia	565 (13)
Specified septicemia	1303 (30)
Bloodstream infection due to CVC	84 (2)
Respiratory infection	1421 (33)
Urinary tract infection	481 (11)
Peritonitis / postoperative infection	435 (10)
Soft tissue infection	365 (8)
Other infection due to CVC/vascular device/graft	300 (7)
Gastrointestinal tract infection	270 (6)
Hepato-biliary infection	134 (3)
Endocarditis	104 (2)
Central nervous system infection	42 (1)
**Infectious agent**	**n (%)**
Escherichia coli spp	258 (6)
Pseudomonas spp	115 (3)
Klebsiella spp	16 (0.4)
Serratia spp	9 (0.2)
Other Gram-negative bacteria	479 (11)
Staphylococcus spp	475 (11)
Streptococcus spp	369 (8)
Anaerobes	70 (2)

## Discussion

The main findings of this study were as follows: in a large population of middle aged adults, the cumulative incidence of sepsis was 3.7% one year after cancer diagnosis and has been increasing over time with about a third of cases developing septic shock. The probability of transitioning to sepsis was about half of the transition probability to death without sepsis at 1 year, but sepsis was associated with a high mortality. Age, CCI, certain cancer sites (hematological malignancies) and metastases at diagnosis were associated with an increased risk of sepsis and death whereas female gender was protective.

Two large studies published about 2 decades ago reported incidence of sepsis in cancer patients in the US of 1465 and 1640 cases/100,000 patients/year respectively [6, 7] whereas a more recent Australian study reported a much higher incidence of 6.4% within a year of cancer diagnosis [10]. Methological differences may account for some of these discrepancies such as the criteria used to define sepsis (ICD-9 versus ICD-10 codes and specific codes included), the inclusion period after cancer diagnosis (1 year versus unlimited) and the use of a competing risk approach versus incidences normalized to population distribution. Moreover, the US studies were based on hospitalization data but authors have reported that cancer was not coded in a significant proportion of cancer patients admitted for sepsis [10], which may affect incidence estimates. The incidence of sepsis we observed was lower than in the Australian study but increased between 2005 and 2014; the inclusion of middle aged patients only may explain this difference as sepsis incidence increases with age [6]. These points being taken into account, our results seem to confirm that the incidence of sepsis in cancer patients has been increasing over time as recently suggested [10] and is anyway well higher than in the general population, where an incidence of about 300 cases per 100,000 persons-year was reported [3]. An aging population may contribute to the rising incidence of sepsis in general but is unlikely to explain our results as we did not include patients 65 years and older; alternative explanations might include more aggressive therapeutic regimen causing profound/prolonged neutropenia or the widespread use of central venous catheters exposing patients to infections; the impact of novel targeted therapy or immunotherapy on the incidence of sepsis would also deserve investigation.

As previously reported [6, 7], we observed that age and male gender were associated with increased incidence of sepsis in cancer patients, and these are also risk factors for sepsis in the general population [9]. The strongest predictors of sepsis in our cohort were metastatic disease at the time of diagnosis and hematological malignancy whereas other cancer sites like breast or genitourinary location were associated with a lower incidence of sepsis. The association between comorbidities and the risk of developing sepsis is well known in the general population [16] and has been reported in cancer patients in terms of binary exposure (non-cancer comorbidities or not) [6]; here we reported more granular data on comorbidities and observed that all components of the Charlson comorbidity index, except for dementia and rheumatic disease, were more frequent in patients who developed sepsis.

How much sepsis contributes to the overall mortality in cancer patients remains unclear, as most studies did not address sepsis as a time dependent variable or did not analyze non cancer-related mortality. In order to tackle this complex issue we used a multistate model, which is a useful model to analyze incidence of intermediate events like sepsis and rates of death [17]. The probability of developing sepsis was higher than the probability of non sepsis-related death within the first 60 days, then the 2 curves crossed and the probability of non-septic death continued to rise sharply whereas the curve almost plateaued for sepsis. Crude hospital mortality of sepsis, severe sepsis and septic shock were 15%, 30% and 46% respectively, in agreement with mortality rates reported in general populations of septic patients [18, 19] and with a recent series of about 2,000 ICU patients with malignancies and sepsis or septic shock who had a 30 day mortality of 40% [8]. In our population, metastatic disease was a predictor of non sepsis-related death as could be expected, but was also strongly associated with transition from sepsis to death: increased treatment limitations may have contributed to this finding, but could not be confirmed as this information was not available in the database. One-year mortality probability was 36% for patients who developed sepsis. We chose to focus on the first year post cancer diagnosis because it is the time period when both incidence of sepsis and standardized mortality ratios are the highest in cancer patients [10, 11].

Our results also provided a snapshot of the sites and pathogen agents responsible for sepsis: in the general population, the most frequent sources of infection leading to severe sepsis are, by order of importance, the respiratory tract, abdomen, urinary tract and bacteremias [20]. In our series, respiratory tract and bloodstream infections were the leading causes, then followed by urinary tract and abdominal infections; the highest proportion of bloodstream infections is likely due to the immunocompromised population, the patients with hematological malignancies being especially exposed [21]. Regarding bloodstream infections, the relative contribution of bacteria involved has changed over the years: Gram-negative rods were the most frequent causative organisms about 5 decades ago, but a shift towards Gram-positive cocci was then reported, likely due to the widespread use of antibacterial prophylaxis and central venous catheters. Some of the most recent studies reported a shifting balance towards gram-negative bacteria again [22]. Overall, all infection sites included, gram-positive and gram-negative bacteria were evenly balanced in our population which is consistent with other reports [23].

Several limitations to this study deserve further discussion: first, the Marketscan database mostly includes patients insured and employed by large companies and does not include patients above the age of 65, so that our results may not be generalizable to older patients and populations with different socio-economic background for example. Second, our study relied for the diagnoses of cancer and sepsis on the accuracy of coding for insurance claims; potential undercoding may have affected our results. Third, detailed data on cancer treatments and remission status are missing; all of the patients received chemotherapy but we were not able to collect the timing of chemotherapy or potential surgical procedures to analyze their impact on sepsis. Finally, we were not able to report data related to sepsis management (ICU admission, timely administration of adequate antibiotics, volume resuscitation or vasopressors), which are not available in the Marketscan database. Likewise, we relied on ICD-9 codes to report the pathogens associated with episodes of sepsis but we cannot exclude that some of these pathogens were contaminants, as exact species and number of positive blood cultures were not available for staphylococcus for instance.

In summary, in a cohort of middle aged adults with a new diagnosis of cancer, at 1 year the cumulative incidence of sepsis was 3.7% and the probability of dying in patients developing sepsis was 36%. Age, male gender, Charlson comorbidity index and metastases at diagnosis were associated with sepsis and mortality. The incidence of sepsis has increased between 2005 and 2014.
